# Survival and Recurrence of Endocarditis following Mechanical vs. Biological Aortic Valve Replacement for Endocarditis in Patients Aged 40 to 65 Years: Data from the INFECT-Registry

**DOI:** 10.3390/jcm13010153

**Published:** 2023-12-27

**Authors:** Antonio Salsano, Michele Di Mauro, Laura Labate, Alessandro Della Corte, Federica Lo Presti, Michele De Bonis, Cinzia Trumello, Mauro Rinaldi, Erik Cura Stura, Guglielmo Actis Dato, Giuseppe Punta, Francesco Nicolini, Davide Carino, Carlo De Vincentiis, Andrea Garatti, Giangiuseppe Cappabianca, Andrea Musazzi, Diego Cugola, Maurizio Merlo, Davide Pacini, Gianluca Folesani, Sandro Sponga, Igor Vendramin, Alberto Pilozzi Casado, Francesco Rosato, Elisa Mikus, Carlo Savini, Francesco Onorati, Giovanni Battista Luciani, Roberto Scrofani, Francesco Epifani, Francesco Musumeci, Antonio Lio, Andrea Colli, Giosuè Falcetta, Salvatore Nicolardi, Salvatore Zaccaria, Enrico Vizzardi, Antonio Pantaleo, Giuseppe Minniti, Emmanuel Villa, Margherita Dalla Tomba, Francesco Pollari, Fabio Barili, Alessandro Parolari, Roberto Lorusso, Francesco Santini

**Affiliations:** 1 Division of Cardiac Surgery, Ospedale Policlinico San Martino, 16132 Genoa, Italy; francesco.santini@unige.it; 2 DISC Department, University of Genoa, 16126 Genoa, Italy; 3 CARIM Maastricht University, 6229 ER Maastricht, The Netherlands; mdimauro1973@gmail.com; 4 Department of Health Sciences (DISSAL), University of Genoa, 16126 Genoa, Italy; laura.labate92@gmail.com; 5 Infectious Diseases Unit, Ospedale Policlinico San Martino-IRCCS, 16132 Genoa, Italy; 6 Unit of Cardiac Surgery, Department of Translational Medical Sciences, Monaldi Hospital, University of Campania “L. Vanvitelli”, 80131 Naples, Italy; aledellacorte@libero.it (A.D.C.); nicopap@hotmail.it (F.L.P.); 7 IRCCS Ospedale San Raffaele, Division of Cardiac Surgery, Università Vita-Salute San Raffaele, 20132 Milan, Italy; debonis.michele@hsr.it (M.D.B.);; 8 Cardiac Surgery, Molinette Hospital, University of Turin, 10124 Turin, Italy; mauro.rinaldi@unito.it (M.R.);; 9 Cardiac Surgery, Mauriziano Hospital, 10128 Turin, Italy; actisdato.g@gmail.com (G.A.D.); gpunta@libero.it (G.P.); 10 Cardiac Surgery, Maggiore University Hospital, University of Parma, 43121 Parma, Italy; francesco.nicolini@unipr.it (F.N.); davide.carino@unipr.it (D.C.); 11 Cardiac Surgery, San Donato IRCCS Hospital, San Donato Milanese, 20097 Milan, Italy; devince@libero.it (C.D.V.); andrea.garatti@grupposandonato.it (A.G.); 12 Cardiac Surgery, University Hospital, 21100 Varese, Italy; giangiuseppe.cappabianca@asst-settelaghi.it (G.C.); andrea.musazzi@asst-settelaghi.it (A.M.); 13 Cardiac Surgery, AO Papa Giovanni XXIII, 24127 Bergamo, Italy; dcugola@asst-pg23.it (D.C.); mmerlo@asst-pg23.it (M.M.); 14 Cardiac Surgery, S. Orsola-Malpighi University Hospital, University of Bologna, 40126 Bologna, Italy; davide.pacini@unibo.it (D.P.); gianluca.folesani@aosp.bo.it (G.F.); 15 Cardiac Surgery, S. Maria Misericordia Hospital, University of Udine, 33100 Udine, Italyigor.vendramin@asufc.sanita.fvg.it (I.V.); 16 Cardiac Surgery, S. Croce Hospital, 12100 Cuneo, Italy; pilozzi.a@ospedale.cuneo.it (A.P.C.); rosato.f@ospedale.cuneo.it (F.R.); fabio.barili@gmail.com (F.B.); 17 GVM Care & Research, Maria Cecilia Hospital, 48033 Cotignola, Italy; elisamikus@yahoo.it (E.M.); csavini@gvmnet.it (C.S.); 18 Cardiac Surgery, University Hospital, University of Verona, 37129 Verona, Italy; francesco.onorati@univr.it (F.O.); giovanni.luciani@univr.it (G.B.L.); 19 Fondazione IRCCS Ca’ Granda, Ospedale Maggiore Policlinico, 20122 Milano, Italy; roberto.scrofani@policlinico.mi.it (R.S.); francesco.epifani1@unimi.it (F.E.); 20 Cardiac Surgery, San Camillo-Forlanini Hospital, 00152 Rome, Italy; fr.musumeci@gmail.com (F.M.); antoniolio@hotmail.it (A.L.); 21 Cardiac Surgery, AO Pisana University Hospital, University of Pisa, 56126 Pisa, Italy; colli.andrea.bcn@gmail.com (A.C.); g.falcetta@ao-pisa.toscana.it (G.F.); 22 Cardiac Surgery, Vito Fazzi Hospital, 73100 Lecce, Italy; salvatore.nicolardi@unipv.it (S.N.); cardiochirurgia.polecce@asl.lecce.it (S.Z.); 23 Cardiology, Spedali Civili Hospital, 25123 Brescia, Italy; vizzardi72@gmail.com; 24 Department of Cardiac Surgery, Azienda ULSS2 Ca’ Foncello Hospital, 31100 Treviso, Italy; antonio.pantaleo@aulss2.veneto.it (A.P.); giuseppe.minniti@aulss2.veneto.it (G.M.); 25 Department of Cardiac Surgery, Poliambulanza Foundation Hospital, 25124 Brescia, Italy; emmanuel.villa@poliambulanza.it (E.V.); margherita.dallatomba@poliambulanza.it (M.D.T.); 26 Cardiac Surgery, Klinikum Nürnberg–Paracelsus Medical University, 90419 Nuremberg, Germany; fpollari@gmail.com; 27 Department of Universitary Cardiac Surgery and Translational Research, IRCCS Policlinico S. Donato, University of Milan, 20122 Milan, Italy; 28 Department of Biomedical Sciences for Health, Università di Milano, 20122 Milan, Italy; 29 Cardio-Thoracic Surgery Department, Heart & Vascular Centre, Maastricht University Medical Centre, 6229 HX Maastricht, The Netherlands; roberto.lorussobs@gmail.com

**Keywords:** endocarditis, aortic valve replacement, prosthetic heart valve

## Abstract

Background: Infective endocarditis (IE) is a serious disease, and in many cases, surgery is necessary. Whether the type of prosthesis implanted for aortic valve replacement (AVR) for IE impacts patient survival is a matter of debate. The aim of the present study is to quantify differences in long-term survival and recurrence of endocarditis AVR for IE according to prosthesis type among patients aged 40 to 65 years. Methods: This was an analysis of the INFECT-REGISTRY. Trends in proportion to the use of mechanical prostheses versus biological ones over time were tested by applying the sieve bootstrapped t-test. Confounders were adjusted using the optimal full-matching propensity score. The difference in overall survival was compared using the Cox model, whereas the differences in recurrence of endocarditis were evaluated using the Gray test. Results: Overall, 4365 patients were diagnosed and operated on for IE from 2000 to 2021. Of these, 549, aged between 40 and 65 years, underwent AVR. A total of 268 (48.8%) received mechanical prostheses, and 281 (51.2%) received biological ones. A significant trend in the reduction of implantation of mechanical vs. biological prostheses was observed during the study period (*p* < 0.0001). Long-term survival was significantly higher among patients receiving a mechanical prosthesis than those receiving a biological prosthesis (hazard ratio [HR] 0.546, 95% CI: 0.322–0.926, *p* = 0.025). Mechanical prostheses were associated with significantly less recurrent endocarditis after AVR than biological prostheses (HR 0.268, 95%CI: 0.077–0.933, *p* = 0.039). Conclusions: The present analysis of the INFECT-REGISTRY shows increased survival and reduced recurrence of endocarditis after a mechanical aortic valve prosthesis implant for IE in middle-aged patients.

## 1. Introduction

Infective endocarditis (IE) is a serious disease, and in many cases, surgery is necessary [1]. From a theoretical point of view, the use of foreign material should be minimized; however, when the native valve is severely compromised, a prosthetic replacement is needed [2]. For the general population with aortic valve disease, the European Society of Cardiology (ESC) suggested surgical implantation of mechanical valves in patients younger than 60 years and bioprostheses in patients over 65 years [3]. However, there is no consensus recommendation on the type of prosthetic valve to use in cases of IE, ref. [2], and few studies have compared mechanical and biological prostheses in IE patients so far, leading to contradictory results [4,5,6,7].

Furthermore, in recent years, middle-aged patients have tended to prefer biological valves to mechanical ones due to concerns related to the use of anticoagulants and the rise of valve-in-valve (ViV) procedures [8].

The aim of the present study is to quantify differences in long-term survival, recurrence of endocarditis, and early postoperative complications after aortic valve replacement (AVR) for IE according to prosthesis type among patients aged 40 to 65.

## 2. Materials and Methods

The ItaliaN Registry For surgical trEatment of valve and prosthesis infeCtive endocardiTis (INFECT-REGISTRY) includes surgically treated patients with IE since February 1979. The Registry is endorsed by the Italian Society for Cardiac Surgery (SICCH) and the Italian Group of Research for Outcome in Cardiac Surgery (GIROC). This registry enrolled patients undergoing isolated surgery for IE at 24 Italian cardiac surgery centers.

The ethical committee approved this study with protocol number 0009040 on 29 January 2015.

The included patients met criteria for a possible or definite IE diagnosis based on modified Duke criteria [9]. Only the first episode of IE recorded for an individual patient was used in the analysis. Patients with definite IE who underwent aortic valve surgery consisting of isolated biological valve replacement with stentless/stented porcine and bovine pericardial valves or isolated mechanical valve replacement with monoleaflet or bileaflet prostheses were included. Exclusion criteria were: age <40 years old and >65 years old; patients undergoing valve repair rather than replacement or receiving a homograft/autograft; patients undergoing aortic root or ascending aortic replacement; patients receiving AVR combined with another valve procedure; and patients whose survival data were missing. A standard case report form was used at all sites to collect data on the index hospitalization. Clinical features, including demographics, comorbidities, pre-existing valvular conditions, and details regarding the current IE episode, including source of acquisition, microbiology, echocardiographic findings, complications, management, and outcome, were collected. All the clinical variables collected in the dataset were defined according to Euroscore [10]. 

From 2000 to 2021, 4365 patients with native valve endocarditis (NVE) or prosthetic valve endocarditis (PVE) were operated on in Italian Cardiac Surgery Centers (Appendix A). Of these, 549 met the inclusion and exclusion criteria mentioned above and represent this study population.

The primary end-points were long-term mortality, defined as any-cause death at follow-up, and recurrence of endocarditis, defined as a diagnosis of IE in a re-admission following discharge after completion of antibiotic therapy recommended for IE [2].

Secondary end-points were: duration of mechanical ventilation (MV), intensive care unit (ICU) stay, hospital stay, permanent pacemaker implantation, atrial fibrillation onset, intra-aortic balloon pump (IABP) use, perioperative stroke, acute kidney injury, reoperation for bleeding, sepsis, multiorgan failure (MOF), and early mortality (in-hospital or 30 days after surgery) due to any cause.

### Statistical Analysis

Categorical data were presented as frequencies and percentages and compared using the Chi-square test or Fisher’s exact test where appropriate. Continuous variables were expressed as means with SD and compared with the t test for normal distributions and expressed as median and interquartile range [IQR] and compared using the two-tailed Mann–Whitney test for non-parametric distributions. No attempt to replace the missing values was made. Trends in proportion to the use of mechanical prostheses versus biological ones over time were tested by applying the sieve bootstrapped *t*-test. Confounding differences in baseline characteristics were addressed using propensity score matching [11]. To calculate the propensity score, a hierarchical logistic regression model was fitted with mechanical implantation as the outcome. Covariates entered into the model include all measured baseline characteristics listed in Table A1 in Appendix A. The area under the receiver operating characteristic curve for this model was 0.704 (Appendix A, Figure A3). The optimal full matching method, a subclassification approach that optimally forms subclasses where one subject is matched to one or more counterparts, has been used [11]. Full matching involves the formation of strata consisting of treated and control subjects and incorporates weights that are derived from the stratification. The baseline characteristics of the patient pairs matched by propensity score were compared using the Kolmogorov–Smirnov test. A standardized difference that was less than 0.1 was deemed indicative of an acceptable balance. For all primary end points, survival curve estimates were derived from the Kaplan–Meier method. For the other primary endpoint recurrence of endocarditis, a competing risk analysis was performed to construct cumulative incidence function curves and to calculate estimates. For all end primary points, marginal Cox proportional hazards regression models with robust sandwich variance estimators were fitted with only prosthesis type entered as a covariate. The difference in overall survival was compared using the Cox model, whereas the differences in recurrence of endocarditis were evaluated using the Gray test. Secondary endpoints were analyzed by regressing the binary outcome on a treatment status indicator using a logistic regression model expressed as odds ratios (OR) and 95% confidence intervals (CI). The model incorporates the weights induced by full matching. A robust, sandwich-type variance estimator has been used to account for the clustering of subjects within strata [12]. The significance level was set for an alpha value of 0.05. Statistical analyses were performed using R software (version 4.3.1; R Foundation for Statistical Computing, Vienna, Austria).

## 3. Results

Among the 549 patients aged between 40 and 65 years and operated on for AVR for IE, 268 (48.8%) received mechanical prostheses and 281 (51.2%) received biological ones.

The baseline and operative characteristics of the overall unadjusted cohort are shown in Table 1. 

Patients who underwent AVR with a mechanical prosthesis were younger (median 52.00, interquartile range (IQR): 45.37–57.56, vs. median 57.00, IQR: 48.74–62.00, *p* < 0.001) and more likely to be female (23.9% vs. 14.2%, *p* = 0.006).

Patients who received biological prosthetic valves were more likely to have had heart failure symptoms (21.0% vs. 10.4%, *p* = 0.001), cardiogenic shock (9.6% vs. 4.9%, *p* = 0.048), and significantly higher orotracheal intubation rates before surgery (10.0% vs. 3.0%, *p* = 0.002).

Leaflet perforation (17.8% vs. 10.8%, *p* = 0.043) and large vegetation (36.3% vs. 45.1%, *p* = 0.043) were more frequent in the bioprosthesis group. Logistic Euroscore was higher (median 6.45 vs. 4.68, *p* = 0.005) in patients receiving AVR with biological prostheses than those treated with mechanical ones, as were cardiopulmonary bypass time (90.94 min vs. 79.26 min, *p* = 0.004) and aortic cross-clamp time (73.39 min vs. 65.53 min, *p* = 0.001).

Streptococci (21.1%) and Staphylococcus aureus (12.9%, Table 2) were the most frequently isolated germs, and culture-negative endocarditis accounted for 30.4% of cases. No significant differences were found in the pathogens responsible for IE in patients undergoing AVR with mechanical vs. biological prostheses.

**Figure A1:**
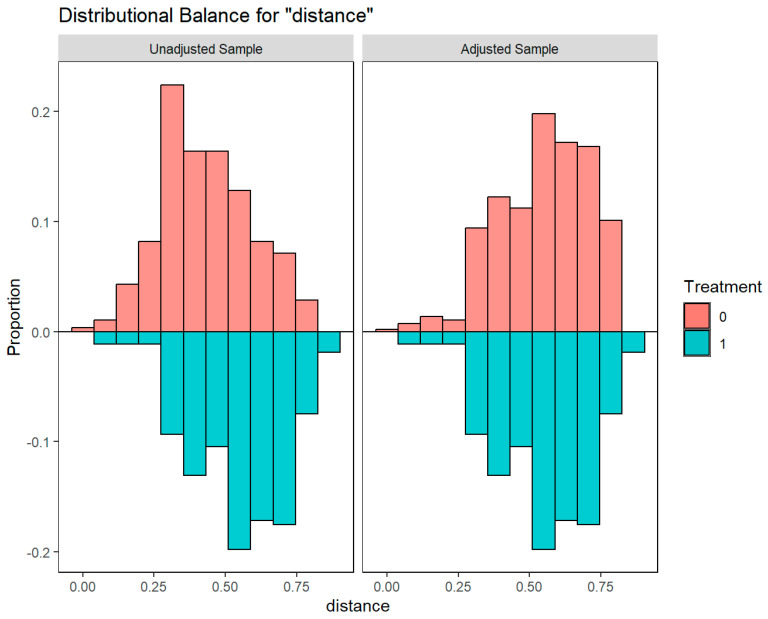
Covariate balance plot for adjusted and unadjusted samples.

**Figure A2:**
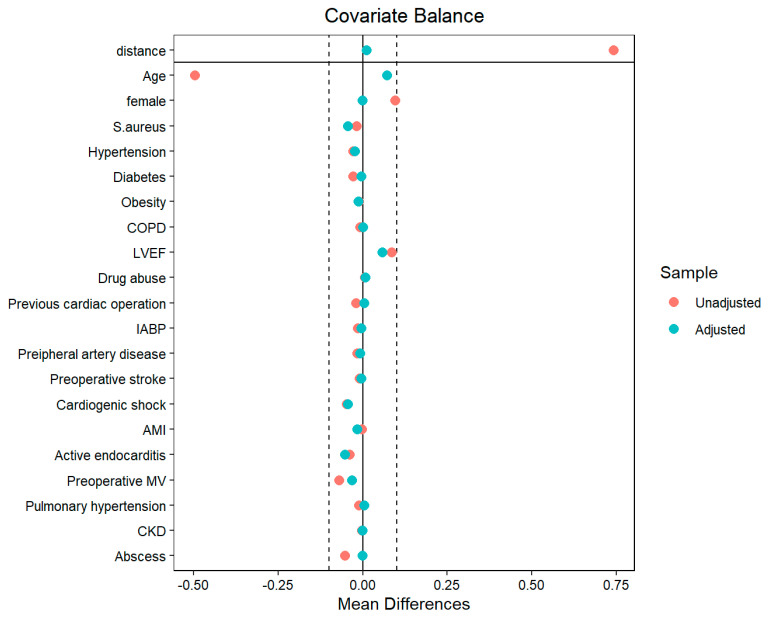
Love plot graphically displaying covariate balance before and after adjusting.

**Figure A3:**
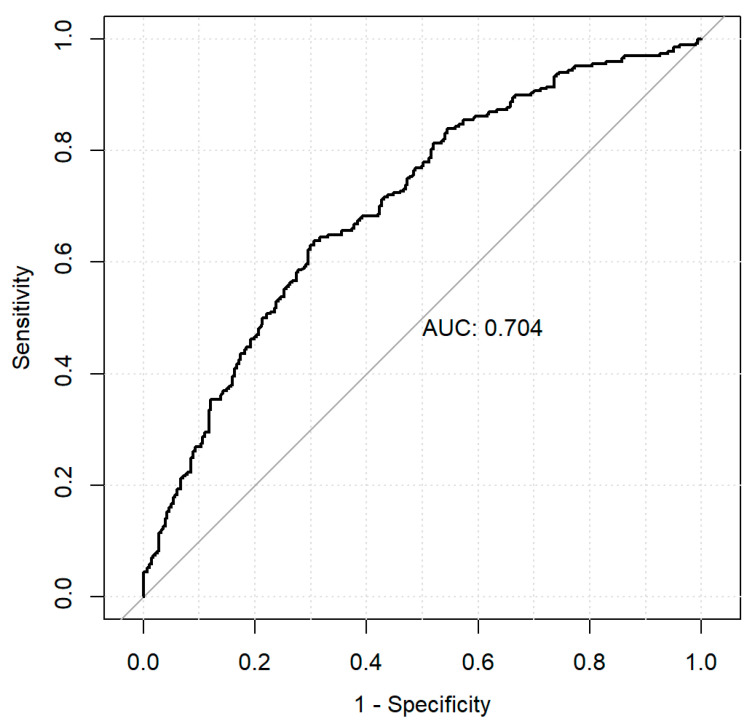
ROC curve of the propensity scores.

An optimal full propensity-score matching produced an adjusted cohort of 268 vs. 281 patients. Age and all baseline comorbidities resulted in a balance between groups (Appendix A, Table A1, and Figure A1 and Figure A2). Patients were followed up for 65 ± 53 months.

### 3.1. Trends in AVR with Mechanical vs. Biological Valves in Middle-Aged Patients

**Figure 1:**
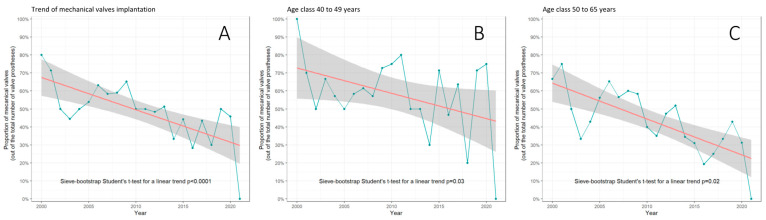
Trend in proportion of the implanted mechanical versus biological prostheses in the overall cohort (**A**), in age class 40–49 years (**B**), and in age class 50–65 years (**C**). Green line: annual fluctuations in percentages; red line: linear trend; gray area: confidence interval around linear trend.

For AVR, mechanical prostheses were less implanted than bioprostheses (268 [48.8%] vs. 281 [51.2%]) among 40-to-65-year-old patients operated on for IE from January 2000 to December 2021. The mechanical versus biological prosthesis ratio (MBPR) during the entire period was 0.95. We observed a shift towards less mechanical AVR (*p* < 0.0001, Figure 1) to reach a MBPR of 0.73 during the last 5 years of the series. A significant trend in reduction of mechanical vs. biological prostheses implantation across time was consistent in age subclasses 40–49 years (*p* = 0.03) and 50–65 years (*p* = 0.02, Figure 1).

### 3.2. Long Term Survival

**Figure 2:**
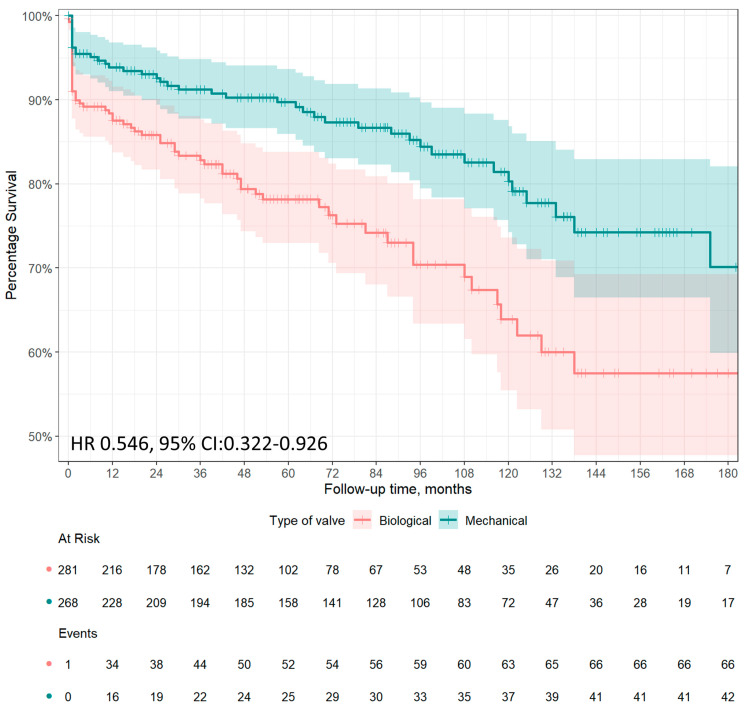
Approximately 15-year survival after AVR for IE in patients aged 40 to 65 years according to prosthetic type: mechanical prostheses (green line) or bioprostheses (red line). Adjusted survival in patients matched by propensity score has been reported as hazard ratio (HR) and 95% confidence interval (CI).

A total of 42 (15.7%) deaths occurred in the mechanical prostheses group and 66 (23.5%) deaths occurred in the bioprostheses group at follow-up; the actuarial survival at 1, 5, 10, and 15 years were 93.9%, 89.7%, 80.3%, and 70.1% in the mechanical prostheses group, and 87.5%, 78.2%, 63.9%, and 57.5% in the bioprostheses group, respectively. A total of 2 (0.7%) deaths during follow-up occurred after major bleeding in patients who received mechanical prostheses, versus 1 (0.4%) death in the bioprostheses group. Only 1 patient treated with bioprothesis died after re-operation for structural valve degeneration (SVD)-related bioprosthetic valve failure, 42 months after the index operation. Among patients matched by propensity score, mid- to long-term survival was significantly higher among patients treated with a mechanical prosthesis than those treated with a biological prosthesis (hazard ratio [HR], 0.546, 95% CI: 0.322–0.926, *p* = 0.025; Figure 2). 

### 3.3. Recurrence of Endocarditis

**Figure 3:**
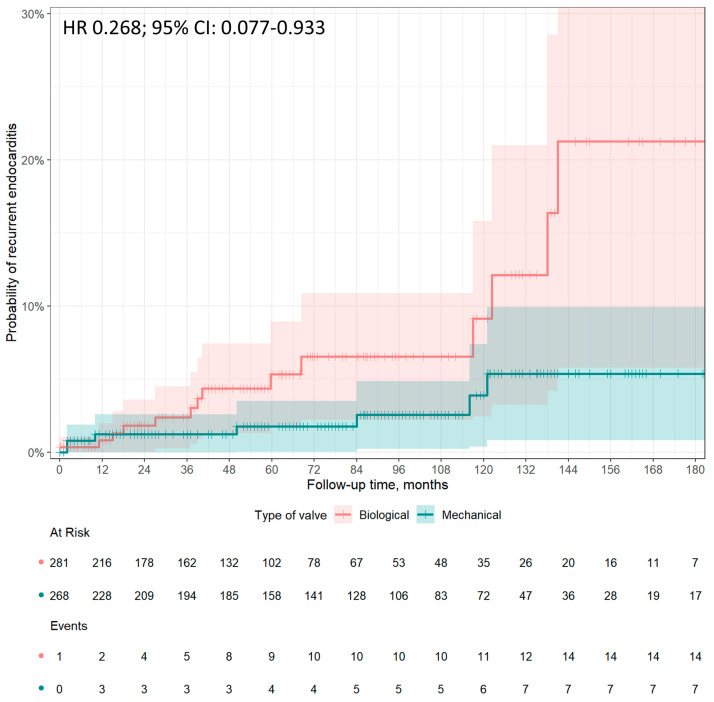
Cumulative incidence of recurrent endocarditis after AVR in IE patients aged 40 to 65 years according to prosthetic type: mechanical prostheses (green line) vs. bioprostheses (red line). Adjusted estimates in patients matched by propensity score have been reported as hazard ratio (HR) and 95% confidence interval (CI).

During the 15 years follow-up, the cumulative incidence of IE recurrence after AVR in our cohort was 3.8% (*N* = 21/549), of which 2.6% (*N* = 7/268) and 5.0% (*N* = 14/281) were in the mechanical and biological protheses groups, respectively (Figure 3). More in depth, the cumulative incidence of recurrence at 1, 5, 10, and 15 years was 2.6%, 3.4%, 7.1%, and 9.5% in the mechanical prostheses group, and 1.9%, 8.6%, 14.6%, and 30.8% in the bioprostheses group, respectively.

In adjusted analysis, prosthesis type was associated with recurrent endocarditis after AVR (HR 0.268, 95% CI: 0.077–0.933, *p* = 0.039, reference mechanical prothesis group, Figure 3). 

A total of 2 out of 7 patients (28.6%) with recurrence of IE on mechanical prostheses and 4 out of 14 patients (28.6%) with recurrence of IE on bioprostheses underwent redo operations. A total of 6 out of 21 (28.6%) patients with a recurrence of IE died during hospitalization.

### 3.4. Early Postoperative Complications 

Early outcomes after AVR for IE in patients aged 40–65 years are shown in Table 3. In brief, early mortality after surgery was 6.2%. Patients with mechanical valves and bioprostheses had comparable early mortality rates (4.1% vs. 8.2%, *p* = 0.07; adjusted OR in matched cohort: 0.480, 95% CI: 0.229–1.005, *p* = 0.052). Postoperative acute kidney injury (2.6% vs. 7.1%, *p* = 0.02, adjusted OR in matched cohort 0.349, 95% CI: 0.145–0.839, *p* = 0.019) and atrial fibrillation (6.3% vs. 15.6%, *p* = 0.001, adjusted OR in matched cohort 0.362, 95% CI: 0.198–0.662, *p* < 0.0001) were significantly less frequent in the mechanical valve group than in the bioprosthesis group. 

Other perioperative complications, including the need for an intra-aortic balloon pump (IABP), stroke, dialysis, reoperation for bleeding, sepsis, multiorgan failure (MOF), pacemaker implantation, mechanical ventilation (MV), intensive care unit (ICU) stay, and hospital stay, did not show significant differences between groups (Table 3). 

## 4. Discussion

The main results stemming from the present analysis of the INFECT-REGISTRY data in middle-aged patients undergoing AVR for IE are the following: (1) patients tended to prefer a biological valve over a mechanical one; (2) patients treated with mechanical prostheses had greater survival and a lower recurrence of endocarditis compared to those treated with biological prostheses at 15 years.

### 4.1. Preference of Prosthetic Valve Types in Middle-Aged Patients

IE is a serious disease associated with high mortality and morbidity rates [1,13]. Surgical debridement and valve repair or replacement are often required [1,13]. The cornerstone of the surgical treatment of an aortic valve IE is represented by AVR with the implantation of a mechanical or biological prosthesis. Homografts are less used and lead to inconsistent results [14,15].

According to the recent ESC guidelines for the management of IE, prosthetic valve selection in IE is influenced by the presence of the following features: recent ischemic stroke, evidence of intracranial bleeding, woman of childbearing age, high likelihood of prolonged mechanical circulatory support, advanced age or frailty, poor or unknown medical compliance, and an expected complicated and prolonged postoperative course [2]. 

Beyond the aforementioned patient-specific characteristics, heart valve disease guidelines recommend a mechanical valve replacement in patients under 60 years and a biological valve implant in patients over 65 years, claiming that patient preference should be taken into account [2,3]. These general considerations may be even more crucial for patients under 65 years of age, since each type of prosthetic valve carries risks and benefits. Mechanical prostheses require long-lasting anticoagulant treatment, with a risk of hemorrhage and thromboembolism, whereas biological valves are associated with a higher risk of failure due to structural valve degeneration (SVD) [16].

In the present study, biological substitutes were predominantly implanted in patients aged between 40 and 65 years. This complies with the study by Caus et al., according to which biological prostheses have recently tended to be preferred over mechanical ones in patients between 50 and 60 years old [8]. In this regard, our study shows a decreasing trend in the preference for mechanical prostheses both for the 40–49 years and for the 50–65 years age classes. The phenomenon seems not to be related to patient comorbidities and has become even more marked in the last 5 years. 

It seems that the concerns about the lifelong anticoagulation required for mechanical valve implantation outweigh the risk of failure of the bioprosthesis due to SVD. The transcatheter-based approach of ViV to treat the failed surgical aortic valve is the primary determinant [7,17]. However, surgeons should be aware that ViV procedures should be planned during the first operation, thus implanting at least a 23 mm diameter valve. For smaller prosthetic sizes, aortic valve regurgitation and severe patient–prosthesis mismatch occur more frequently in ViV than in surgical aortic valve re-replacement [17].

### 4.2. Perioperative Outcomes, Long-Term Survival, and Recurrence of IE after AVR for IE in Middle-Aged Patients

Studies comparing outcomes of different prosthetic valve types in AVR for IE are scarce and mostly not focused on middle-aged patients [4,5,6,7,18].

Our study showed no significant difference in early mortality among patients treated with mechanical vs. biological prostheses. The overall early mortality rate was 6.2% after AVR for IE, consistent with mortality rates reported in the literature [1,18,19]. In our series, patients receiving a mechanical prosthesis had a significantly lower occurrence of atrial fibrillation and acute kidney injury. It was confirmed after adjustment for the propensity score. Since these results after AVR for IE have not been reported in the literature, they need further studies to be confirmed.

Contradictory results have been reported for long-term survival in patients undergoing AVR with mechanical prostheses versus bioprostheses for IE [4,5,6,19,20,21]. A propensity-weighted observational study on 395 patients with left-sided endocarditis in patients over 18 years of age showed lower mortality at 10 years, 51.4 ± 6.3% versus 73.7 ± 12.0%, for the mechanical valve group vs. biological valve group. This study missed analyzing subgroups for aortic valve disease or age stratification [21]. Said et al. and Musci et al., in their retrospective, unadjusted studies, showed no difference in late survival after the implantation of biological or mechanical prostheses [22,23]. Nguyen et al. found that patients <65 years of age receiving a bioprosthetic aortic valve for IE had a significantly increased risk of 5-year mortality compared with those receiving a mechanical valve [20]. Toyoda et al. found no difference in 12-year survival among patients undergoing AVR for IE with either a mechanical or a biological prosthesis. Toyoda et al. found no difference in 12-year survival between patients who underwent AVR for IE with a mechanical or biologic prosthesis, and age did not influence the results [6]. In a large retrospective observational study, Delahaye et al. found a 1-year mortality rate of 28.4% in the bioprostheses group and 19.7% in the mechanical prostheses group. The use of bioprostheses was independently associated with 1-year mortality, and the hazard ratio was significantly higher in patients under 65 years of age [5]. Kytö et al. selected 213 patients aged 16 to 70 years who underwent first-time AVR for IE with a mechanical or biological prosthetic valve in Finland between 2004 and 2014. A significantly lower 5-year mortality with mechanical prosthesis (19% vs. 35%), with a HR of 0.47, 95% CI: 0.23–0.92, was reported. The association was not modified by age <60 years versus 60–70 years [4]. Our results are consistent with previous findings. In patients aged 40 to 65 years of age, survival rates at 1, 5, 10, and 15 years were 93.9%, 89.7%, 80.3%, and 70.1% in the mechanical prostheses group, and 87.5%, 78.2%, 63.9%, and 57.5% in the bioprostheses group. 

Mid- to long-term survival was significantly higher even in propensity-matched patients treated with a mechanical prosthesis than in those treated with a biological prosthesis. In the absence of randomized controlled trials in middle-aged IE patients undergoing AVR, it is uncertain what the real cause of this difference in long-term survival is. It may be, at least in part, due to the need for reoperation for patients with bioprostheses. According to Chiang et al., 12.1% of patients who received biological prostheses subsequently underwent aortic valve reoperation for SVD-related bioprosthetic valve failure, compared with 6.9% of the mechanical prostheses group. A 30-day mortality rate after reoperation was 9.0%; however, efforts can be made to reduce mortality [16]. High-volume valve centers report early mortality rates of 3% to 5% for redo AVR; notably, it may be no different from mortality for the index AVR [24]. Furthermore, transcatheter ViV procedures have emerged as an alternative to redo valve surgery in high-risk patients [25]. SVD includes a wide range of valve abnormalities such as calcification, leaflet tear, stent creep, and suture line disruption of components of the bioprosthesis, resulting in valve stenosis or incompetence, and finally, biological valve failure [26]. It is likely that the smaller effective valve area of prosthetic valves compared with normally functioning native valves produces a certain degree of left ventricular overload that could affect ventricular function over time, causing symptoms and ultimately reducing survival. A correlation between SVD and IE has been hypothesized [27]. Conversely, SVD could mutually accelerate SVD. The assumption, which requires confirmation, is that platelet–fibrin complex formation on damaged endothelium and subsequent establishment of non-bacterial thrombotic endocarditis is an essential component of bacterial adherence and eventual infiltration [27]. There is no demonstration of the above; however, our study has shown that patients aged between 40 and 65 years undergoing AVR for IE have less recurrence of endocarditis at follow-up after the implantation of a mechanical prosthesis. Comparable results are reported by the few studies published in the literature on the recurrence of IE in patients undergoing valve replacement with biological vs. mechanical prostheses. According to Rubino et al., the need for valve reoperation due to recurrent PVE occurred in 7.3% of patients after mechanical valve replacement and in 17.3% after bioprosthetic valve replacement [21]. Havers-Borgersen et al. showed that the risk of IE recurrence was significantly higher among patients with biological prostheses than among those receiving mechanical prostheses (6.3% vs. 4.6%). On the contrary, Toyoda et al. failed to show a difference between the prosthesis types after 12 years of follow-up in IE patients [6]. Again, randomized studies on the topic are needed to confirm or refute these findings.

### 4.3. Infective Endocarditis Registries

The relatively low incidence of IE, the management in non-referral centers for many patients, and the need for urgent surgery justifies the paucity of randomized controlled studies on IE [1]. Indeed, observational data collected prospectively in IE registries, mostly nationwide, may provide information not disclosed by randomized trials [28]. In particular, we refer to the epidemiology, clinical presentation, natural history of the disease, and prognosis after treatment [28].

The most popular registries on the topic are the International Collaboration on Endocarditis, the European Society of Cardiology (ESC) EUROpean ENDOcarditis (EURO-ENDO) registry [1], particularly oriented to cardiological features, the International Collaboration on Endocarditis (ICE) [5], the Society of Thoracic Surgeons-Infective Endocarditis Cohort (STS-IE) [29], the French Association pour l’Etude et la Prevention de l’Endocardite Infectieuse (AEPEI) cohort [20], the Swedish Register for Infective Endocarditis [30], and the Spanish Grupos de Apoyo al Manejo de la Endocarditis en Espana (GAMES) [31]. 

The present study from the Italian INFECT registry provides new insights on survival and recurrence of IE after mechanical versus biological AVR for IE in middle-aged patients. Noteworthy, the 15-year survival benefit reported in favor of mechanical prostheses over biological prostheses is in line with the 1-year and 5-year results from the ICE and AEPEI registries, respectively [5,20]. We believe that the INFECT registry will contribute to filling surgical knowledge gaps in order to improve the clinical outcomes of IE patients in the near future.

### 4.4. Study Limitations

Our study has some limitations. The first is the retrospective observational nature of the analysis, which may imply differences in surgical and medical treatment over a 21-year period. In order to minimize the differences between the analyzed groups, optimal full-propensity score matching was applied. Second, this is an analysis of a registry not primarily dedicated to the assessment of the objectives of the present study. For instance, we do not have a complete follow-up on reoperations for biological valves for SVD. Similarly, we are not aware of all major bleeding events related to mechanical valves. However, information on reoperations due to SVD-related bioprosthetic valve failure or recurrent IE and on deaths after reoperation or major bleeding is included in the registry. Third, standards of treatment could be different in certain centers. Finally, several guidelines were published during the study period, so we decided to cite just the current one.

## 5. Conclusions

Among propensity-matched patients aged 40 to 65 years who underwent AVR for IE, the implantation of a mechanical prosthesis was associated with significant survival benefits and a reduction in IE recurrence. These findings suggest that mechanical valves are a reasonable choice for patients aged 40 to 65 years in this setting. Nonetheless, real-world data show that the majority of patients nowadays prefer a biological prosthesis.

## Figures and Tables

**Table 1:** Baseline and operative characteristics of patients, aged between 40 and 65 years, with IE treated with mechanical versus biological aortic valve prostheses.

Variables	Mechanical Valve (*N* = 268)	Biological Valve (*N* = 281)	*p* Value
Age (years, median [IQR])	52.00 [45.37, 57.56]	57.00 [48.74, 62.00]	<0.001
Female gender, *n* (%)	64 (23.9)	40 (14.2)	0.006
Hypertension, *n* (%)	64 (23.9)	75 (26.7)	0.510
Diabetes, *n* (%)	23 (8.6)	32 (11.4)	0.341
Obesity, *n* (%)	18 (6.7)	22 (7.8)	0.736
COPD, *n* (%)	13 (4.9)	16 (5.7)	0.802
Drug abuse, *n* (%)	14 (5.2)	13 (4.6)	0.900
Previous cardiac surgery, *n* (%)	8 (3.0)	14 (5.0)	0.330
LVEF (%, mean (SD))	54.94 (8.45)	54.22 (9.47)	0.346
Peripheral arteriopathy, *n* (%)	8 (3.0)	13 (4.6)	0.436
Preoperative stroke, *n* (%)	30 (11.2)	34 (12.1)	0.843
Heart failure, *n* (%)	28 (10.4)	59 (21.0)	0.001
Cardiogenic shock, *n* (%)	13 (4.9)	27 (9.6)	0.048
Acute myocardial infarction (within 90 days), *n* (%)	3 (1.1)	4 (1.4)	1.000
Active endocarditis, *n* (%)	205 (76.5)	226 (80.4)	0.309
Preoperative MV, *n* (%)	8 (3.0)	28 (10.0)	0.002
Pulmonary hypertension (<50 mmHg), *n* (%)	15 (5.6)	19 (6.8)	0.697
CKD, *n* (%)	26 (9.7)	28 (10.0)	1.000
Dialysis, *n* (%)	11 (4.1)	11 (3.9)	1.000
IABP, *n* (%)	1 (0.4)	5 (1.8)	0.241
Preoperative pacemaker implantation, *n* (%)	2 (0.7)	6 (2.1)	0.317
NVE, *n* (%)	267 (99.6)	281 (100)	1.000
PVE, *n* (%)	1 (0.4)	0 (0.0)	1.000
Abscess, n (%)	27 (10.1)	43 (15.3)	0.088
Large vegetation (>1 cm), *n* (%)	121 (45.1)	102 (36.3)	0.043
Leaflet perforation, *n* (%)	29 (10.8)	50 (17.8)	0.027
Paravalvular leak, *n* (%)	1 (0.4)	1 (0.4)	1.000
CABG, *n* (%)			0.367
0	257 (95.9)	265 (94.3)	
1	9 (3.3)	12 (4.3)	
2	1 (0.4)	4 (1.4)	
≥3	1 (0.4)	0 (0.0)	
Logistic Euroscore (median [IQR])	4.68 [2.78, 8.90]	6.45 [3.62, 13.61]	0.005
Cardiopulmonary bypass time (min, mean (SD))	79.26 (33.47)	90.94 (49.47)	0.004
Aortic cross-clamp time (min, mean (SD))	63.53 (23.62)	73.39 (34.97)	0.001

CABG, coronary artery bypass grafting; CKD, chronic kidney disease; COPD, chronic obstructive pulmonary disease; IABP, intra-aortic balloon pump; LVEF, left ventricular ejection fraction; MV, mechanical ventilation; NVE, native valve endocarditis; PVE, prosthetic valve endocarditis.

**Table 2:** Causative agents of IE.

Causative Agent	Mechanical Valve (*N* = 268)	Biological Valve (*N* = 281)	*p* Value
Culture negative endocarditis, *n* (%)	84 (31.3)	83 (29.5)	0.714
Streptococci, *n* (%)	55 (20.5)	61 (21.7)	0.814
Staphylococcus aureus, *n* (%)	34 (12.7)	37 (13.5)	0.600
Viridans group streptococci *, *n* (%)	30 (11.2)	28 (10.0)	0.742
Coagulase-negative staphylococci *, *n* (%)	23 (8.6)	20 (7.1)	0.632
Gram-negative bacteria (non HACEK), *n* (%)	5 (1.9)	9 (3.2)	0.470
Nutritionally variant streptococci *, *n* (%)	2 (0.7)	3 (1.1)	1.000
*Candida* spp., *n* (%)	2 (0.7)	1 (0.4)	0.967
HACEK group *, *n* (%)	1 (0.4)	0 (0.0)	0.981
Enterococcus faecalis, *n* (%)	1 (0.004)	1 (0.004)	1.000
Other organisms, *n* (%)	32 (11.9)	37 (13.2)	0.591

All etiological diagnoses of surgically treated IE were made through cultures. No diagnosis was made through molecular analysis of cultures. No diagnosis was made through molecular methods, and there were no serological diagnoses of *Coxiella burnetii* endocarditis in operated patients. * Viridans group streptococci included *Streptococcus bovis* group, *Streptococcus pyogenes*, *Streptococcus penumoniae*; Coagulase-negative staphylococci included *Staphylococcus epidermidis*, *Staphylococcus hominis*, *Staphylococcus capitis*, *Staphylococcus haemolyticus*; Nutritionally variant streptococci included *Abiotrophia defectiva*, *Granulicatella adiacens*; HACEK group included *Haemophilus* species, *Aggregatibacter actinomycetemcomitans*, *Cardiobacterium hominis*, *Eikenella corrodens*, *Kingella kingae*.

**Table 3:** Early outcomes in overall series and matched cohort of patients, aged 40 to 65 years.

Variables	Overall Series			Estimation in Matched Cohort ^§^		
	Mechanical Valve (*N* = 268)	Biological Valve (*N* = 281)	*p* Value	Odds Ratio	95% CI	*p* Value
Early mortality, *n* (%)	11 (4.1)	23 (8.2)	0.071	0.480	0.229–1.005	0.052
Sepsis, *n* (%)	9 (3.4)	11 (3.9)	0.898	0.849	0.346–2.084	0.722
MOF, *n* (%)	1 (0.4)	7 (2.6)	0.088	0.147	0.018–1.207	0.074
Reoperation for bleeding, *n* (%)	7 (2.6)	11 (3.9)	0.537	0.658	0.251–1.724	0.395
Pacemaker implantation, *n* (%)	4 (1.5)	7 (2.5)	0.592	0.591	0.171–2.042	0.406
Atrial fibrillation, *n* (%)	16 (6.3)	42 (15.6)	0.001	0.362	0.198–0.662	<0.0001
IABP, *n* (%)	3 (1.1)	6 (2.1)	0.544	0.517	0.128–2.088	0.354
Stroke, *n* (%)	6 (2.2)	3 (1.1)	0.460	0.712	0.523–8.541	0.293
Acute kidney injury, *n* (%)	7 (2.6)	20 (7.1)	0.024	0.349	0.145–0.839	0.019
Dialysis, *n* (%)	1 (0.4)	4 (1.5)	0.401	0.261	0.029–2.350	0.231
MV (hours, mean (SD))	23.74 (76.34)	33.56 (82.75)	0.315	−9.819 ^§^	9.763 ^§^	0.315 ^§^
ICU stay (days, median [IQR])	2.00 [1.00, 3.00]	4.00 [2.00, 9.00]	<0.001	−17.700 ^§^	13.599 ^§^	0.194 ^§^
Hospital stay (median [IQR])	12.00 [8.50, 17.00]	12.50 [8.00, 19.00]	0.356	−2.041 ^§^	1.367 ^§^	0.136 ^§^

CI, confidence interval; IABP, intra-aortic balloon pump; ICU, intensive care unit; IQR: interquartile range; MOF, multiorgan failure; MV, mechanical ventilation; Reference for the events: Mechanical valve cohort. ^§^ Least squares regression for continuous dependent variables has been expressed as standard regression coefficient, standard error, and *p* value.

## Data Availability

The Italian Group of Research for Outcome in Cardiac Surgery (GIROC) for the Italian Society for Cardiac Surgery (SICCH) had full access to all of the data in this study and took responsibility for the integrity of the data and the accuracy of the data analysis. The data underlying this article will be shared, on reasonable request, with the corresponding author.

## Appendix A

**Table A1:** Covariate balance analyses in propensity matched samples for patients with biological vs. mechanical valve prostheses.

Variable	Type	Unadjusted Sample							Adjusted Sample						
		Biological Valve 281 pts		Mechanical Valve 268 pts		Balance Measures			Biological Valve 281 pts		Mechanical Valve 268 pts		Balance Measures		
		Mean	Standard Deviation	Mean	Standard Deviation	Mean Difference	Variance Ratio	KS	Mean	Standard Deviation	Mean	Standard Deviation	Mean Difference	Variance Ratio	KS
Distance		0.4302	0.1604	0.5489	0.1602	0.7407	0.9974	0.3320	0.5471	0.1577	0.5489	0.1602	0.0109	1.0319	0.0448
Age	Contin.	55.2723	7.3493	51.7304	7.1413	−0.4960	0.9442	0.2355	51.2162	7.1479	51.7304	7.1413	0.0720	0.9982	0.1008
Female gender	Binary	0.1423	.	0.2388	.	0.0965	.	0.0965	0.2397	.	0.2388	.	−0.0009	.	0.0009
*S. aureus*	Binary	0.1459	.	0.1269	.	-0.0190	.	0.0190	0.1712	.	0.1269	.	−0.0443	.	0.0443
Hypertension	Binary	0.2669	.	0.2388	.	−0.0281	.	0.0281	0.2618	.	0.2388	.	−0.0230	.	0.0230
Diabetes	Binary	0.1139	.	0.0858	.	−0.0281	.	0.0281	0.0898	.	0.0858	.	−0.0040	.	0.0040
Obesity	Binary	0.0783	.	0.0672	.	−0.0111	.	0.0111	0.0805	.	0.0672	.	−0.0133	.	0.0133
COPD	Binary	0.0569	.	0.0485	.	−0.0084	.	0.0084	0.0483	.	0.0485	.	0.0002	.	0.0002
LVEF	Contin.	54.2206	9.4673	54.9440	8.4539	0.0856	0.7974	0.0925	54.4560	9.6088	54.9440	8.4539	0.0577	0.7741	0.0726
Drug abuse	Binary	0.0463	.	0.0522	.	0.0060	.	0.0060	0.0441	.	0.0522	.	0.0082	.	0.0082
Previous cardiac operation	Binary	0.0498	.	0.0299	.	−0.0200	.	0.0200	0.0252	.	0.0299	.	0.0046	.	0.0046
IABP	Binary	0.0178	.	0.0037	.	−0.0141	.	0.0141	0.0083	.	0.0037	.	−0.0045	.	0.0045
Peripheral artery disease	Binary	0.0463	.	0.0299	.	−0.0164	.	0.0164	0.0386	.	0.0299	.	−0.0087	.	0.0087
Preoperative stroke	Binary	0.1210	.	0.1119	.	−0.0091	.	0.0091	0.1172	.	0.1119	.	−0.0052	.	0.0052
Cardiogenic shock	Binary	0.0961	.	0.0485	.	−0.0476	.	0.0476	0.0920	.	0.0485	.	−0.0434	.	0.0434
AMI	Binary	0.0142	.	0.0112	.	−0.0030	.	0.0030	0.0270	.	0.0112	.	−0.0158	.	0.0158
Active endocarditis	Binary	0.8043	.	0.7649	.	−0.0393	.	0.0393	0.8177	.	0.7649	.	−0.0527	.	0.0527
Preoperative MV	Binary	0.0996	.	0.0299	.	−0.0698	.	0.0698	0.0611	.	0.0299	.	−0.0312	.	0.0312
Pulmonary hypertension	Binary	0.0676	.	0.0560	.	−0.0116	.	0.0116	0.0526	.	0.0560	.	0.0034	.	0.0034
CKD	Binary	0.0996	.	0.0970	.	−0.0026	.	0.0026	0.0980	.	0.0970	.	−0.0010	.	0.0010
Abscess	Binary	0.1530	.	0.1007	.	−0.0523	.	0.0523	0.1016	.	0.1007	.	−0.0009	.	0.0009

AMI, acute myocardial infarction; CKD, chronic kidney disease; COPD, chronic obstructive pulmonary disease; IABP, intra-aortic balloon pump; KS, Kolmogorov–Smirnov test; LVEF, left ventricular ejection fraction; MV, mechanical ventilation. For further information on balance measures, see the reference: Austin PC, Stuart EA. Moving towards best practice when using inverse probability of treatment weighting (IPTW) using the propensity score to estimate causal treatment effects in observational studies [32].

## References

[1] Citro R., Chan K.-L., Miglioranza M.H., Laroche C., Benvenga R.M., Furnaz S., Magne J., Olmos C., Paelinck B.P., Pasquet A. (2022). Clinical profile and outcome of recurrent infective endocarditis. Heart.

[2] Delgado V., Marsan N.A., de Waha S., Bonaros N., Brida M., Burri H., Caselli S., Doenst T., Ederhy S., Erba P.A. (2023). 2023 ESC Guidelines for the management of endocarditis. Eur. Heart J..

[3] Vahanian A., Beyersdorf F., Praz F., Milojevic M., Baldus S., Bauersachs J., Capodanno D., Conradi L., De Bonis M., De Paulis R. (2022). 2021 ESC/EACTS Guidelines for the management of valvular heart disease. Eur. Heart J..

[4] Kytö V., Ahtela E., Sipilä J., Rautava P., Gunn J. (2019). Mechanical versus biological valve prosthesis for surgical aortic valve replacement in patients with infective endocarditis. Interact. Cardiovasc. Thorac. Surg..

[5] Delahaye F., Chu V.H., Altclas J., Barsic B., Delahaye A., Freiberger T., Gordon D.L., Hannan M.M., Hoen B., Kanj S.S. (2015). One-year outcome following biological or mechanical valve replacement for infective endocarditis. Int. J. Cardiol..

[6] Toyoda N., Itagaki S., Tannous H., Egorova N.N., Chikwe J. (2018). Bioprosthetic Versus Mechanical Valve Replacement for Infective Endocarditis: Focus on Recurrence Rates. Ann. Thorac. Surg..

[7] Havers-Borgersen E., Butt J.H., Østergaard L., Bundgaard H., Smerup M., Bruun N.E., Gislason G.H., Torp-Pedersen C., Køber L., Fosbøl E.L. (2020). Recurrent infective endocarditis versus first-time infective endocarditis after heart valve surgery. Clin. Res. Cardiol..

[8] Caus T., Chabry Y., Nader J., Fusellier J.F., De Brux J.L., for the EpiCard investigators (2023). Trends in SAVR with biological vs. mechanical valves in middle-aged patients: Results from a French large multi-centric survey. Front. Cardiovasc. Med..

[9] Li J.S., Sexton D.J., Mick N., Nettles R., Fowler V.G., Ryan T., Bashore T., Corey G.R. (2000). Proposed Modifications to the Duke Criteria for the Diagnosis of Infective Endocarditis. Clin. Infect. Dis..

[10] Nashef S., Roques F., Michel P., Gauducheau E., Lemeshow S., Salamon R. (1999). European system for cardiac operative risk evaluation (EuroSCORE). Eur. J. Cardio-Thoracic Surg..

[11] Zhao Q.-Y., Luo J.-C., Su Y., Zhang Y.-J., Tu G.-W., Luo Z. (2021). Propensity score matching with R: Conventional methods and new features. Ann. Transl. Med..

[12] Austin P.C., A Stuart E. (2015). Estimating the effect of treatment on binary outcomes using full matching on the propensity score. Stat. Methods Med. Res..

[13] Kong W.K., Salsano A., Giacobbe D.R., A Popescu B., Laroche C., Duval X., Schueler R., Moreo A., Colonna P., Piper C. (2022). Outcomes of culture-negative vs. culture-positive infective endocarditis: The ESC-EORP EURO-ENDO registry. Eur. Heart J..

[14] Salsano A., Giacobbe D.R., Sportelli E., Olivieri G.M., Natali R., Prevosto M., Del Bono V., Viscoli C., Santini F. (2018). Aortic cross-clamp time and cardiopulmonary bypass time: Prognostic implications in patients operated on for infective endocarditis. Interact. Cardiovasc. Thorac. Surg..

[15] Nappi F., Nenna A., Spadaccio C., Singh S.S.A., Almazil A., Acar C. (2023). The Use of the Cryopreserved Aortic Homograft for Aortic Valve Replacement: Is It Still an Option?. J. Cardiovasc. Dev. Dis..

[16] Chiang Y.P., Chikwe J., Moskowitz A.J., Itagaki S., Adams D.H., Egorova N.N. (2014). Survival and Long-term Outcomes Following Bioprosthetic vs. Mechanical Aortic Valve Replacement in Patients Aged 50 to 69 Years. JAMA.

[17] Raschpichler M., de Waha S., Holzhey D., Schwarzer G., Flint N., Kaewkes D., Bräuchle P.T., Dvir D., Makkar R., Ailawadi G. (2022). Valve-in-Valve Transcatheter Aortic Valve Replacement Versus Redo Surgical Aortic Valve Replacement for Failed Surgical Aortic Bioprostheses: A Systematic Review and Meta-Analysis. J. Am. Heart Assoc..

[18] Savage E.B., Saha-Chaudhuri P., Asher C.R., Brennan J.M., Gammie J.S. (2014). Outcomes and Prosthesis Choice for Active Aortic Valve Infective Endocarditis: Analysis of The Society of Thoracic Surgeons Adult Cardiac Surgery Database. Ann. Thorac. Surg..

[19] Formica F., Maestri F., Gripshi F., Gallingani A., Grossi S., Nicolini F. (2021). Long-Term Outcome of Mechanical and Biological Prostheses in Patients with Left-Side Infective Endocarditis: A Systematic Review and Meta-Analysis. J. Clin. Med..

[20] Nguyen D.T., Delahaye F., Obadia J.-F., Duval X., Selton-Suty C., Carteaux J.-P., Hoen B., Alla F., for the AEPEI Study Group (2010). Aortic valve replacement for active infective endocarditis: 5-year survival comparison of bioprostheses, homografts and mechanical prostheses. Eur. J. Cardio-Thorac. Surg..

[21] Rubino A.S., Della Ratta E.E., Galbiati D., Ashurov R., Galgano V.L., Montella A.P., De Feo M., Della Corte A. (2021). Can prosthesis type influence the recurrence of infective endocar-ditis after surgery for native valve endocarditis? A propensity weighted comparison. Eur. J. Cardiothorac. Surg..

[22] Said S.M., Abdelsattar Z.M., Schaff H.V., Greason K.L., Daly R.C., Pochettino A., Joyce L.D., Dearani J.A. (2018). Outcomes of surgery for infective endocarditis: A single-centre experience of 801 patients. Eur. J. Cardio-Thorac. Surg..

[23] Moon M.R., Miller D.C., Moore K.A., Oyer P.E., Mitchell R.S., Robbins R.C., Stinson E.B., Shumway N.E., Reitz B.A. (2001). Treatment of endocarditis with valve replacement: The question of tissue versus mechanical prosthesis. Ann. Thorac. Surg..

[24] Potter D.D., Sundt T.M., Zehr K.J., Dearani J.A., Daly R.C., Mullany C.J., McGregor C.G., Puga F.J., Schaff H.V., Orszulak T.A. (2005). Operative risk of reoperative aortic valve replacement. J. Thorac. Cardiovasc. Surg..

[25] Attias D., Nejjari M., Nappi F., Dreyfus J., Eleid M.F., Rihal C.S. (2018). How to treat severe symptomatic structural valve deterioration of aortic surgical bioprosthesis: Transcatheter valve-in-valve implantation or redo valve surgery?. Eur. J. Cardio-Thorac. Surg..

[26] Flameng W., Herregods M.-C., Vercalsteren M., Herijgers P., Bogaerts K., Meuris B. (2010). Prosthesis-Patient Mismatch Predicts Structural Valve Degeneration in Bioprosthetic Heart Valves. Circulation.

[27] Kossar A.P., George I., Gordon R., Ferrari G. (2019). Bacterial infiltration and bioprosthetic valve failure: Emerging diagnostics for emerging therapies. J. Thorac. Cardiovasc. Surg..

[28] Escolà-Vergé L., Ribera A., Ferreira-González I., Pericàs J.M., Fernández-Hidalgo N. (2022). Strengths and limitations of patient registries in infective endocarditis. Clin. Microbiol. Infect..

[29] Gaca J.G., Sheng S., Daneshmand M.A., O’brien S., Rankin J.S., Brennan J.M., Hughes G.C., Glower D.D., Gammie J.S., Smith P.K. (2011). Outcomes for endocarditis surgery in North America: A simplified risk scoring system. J. Thorac. Cardiovasc. Surg..

[30] Bläckberg A., Morenius C., Olaison L., Berge A., Rasmussen M. (2021). Infective endocarditis caused by HACEK group bacteria—A registry-based comparative study. Eur. J. Clin. Microbiol. Infect. Dis..

[31] Muñoz P., Kestler M., De Alarcon A., Miro J.M., Bermejo J., Rodríguez-Abella H., Fariñas M.C., Belaustegui M.C., Mestres C., Llinares P. (2015). Current Epidemiology and Outcome of Infective Endocarditis: A Multicenter, Prospective, Cohort Study. Medicine.

[32] Austin P.C., Stuart E.A. (2015). Moving towards best practice when using inverse probability of treatment weighting (IPTW) using the propensity score to estimate causal treatment effects in observational studies. Stat. Med..

